# Knowledge-attitude-practice of pneumoconiosis prevention and control among Chinese university students: a cross-sectional study

**DOI:** 10.3389/fpubh.2026.1816856

**Published:** 2026-06-24

**Authors:** Guomei Gong, Nan Huang, Zhanyou Huang, Xun Zhang, Huiting Wang

**Affiliations:** 1 Department of Humanities and Bilingual Nursing, Quanzhou Medical College, Quanzhou, China; 2 Department of Laboratory Medicine, Quanzhou Medical College, Quanzhou, China; 3 Department of Occupational Health, Quanzhou Center for Disease Control and Prevention, Quanzhou, China; 4 School of Artificial Intelligence and Computer Science, Hubei Normal University, Huangshi, China; 5 College of Medicine and Health Science, Wuhan Polytechnic University, Wuhan, China

**Keywords:** cross-sectional, knowledge-attitude-practice, pneumoconiosis, prevention and control, university student

## Abstract

**Background:**

Pneumoconiosis remains a significant public health issue. Given its substantial harm to patients and society, effective prevention is crucial. The study aimed to investigate the current status systematically and the influencing factors of knowledge, attitudes, and practices regarding the prevention and control of pneumoconiosis among Chinese college students, thereby providing evidence-based recommendations for targeted intervention strategies.

**Methods:**

A cross-sectional online study was conducted based on the Knowledge-Attitude-Practice (KAP) model between September and October 2025. We conducted a nationwide, anonymous survey using stratified random sampling by educational level and geographic region, involving 1,795 students from 84 universities across 33 Chinese provinces. The questionnaire assessed demographic characteristics, as well as knowledge, attitudes, and practices related to the prevention and control of pneumoconiosis. Multiple linear regression analyses were performed to evaluate the impact of demographic factors on the KAP model.

**Results:**

Students demonstrated a moderate overall KAP level across the assessed domains (overall KAP score: 66.93 ± 10.76) with limited knowledge (61.79 ± 16.75) and practical engagement (67.65 ± 13.53), but relatively positive attitudes (72.45 ± 9.92). Non-Han status (β = −0.072), Non-Medical disciplines (β = −0.062), residence in the Northern (β = −0.066), Northwestern (β = −0.073), Southwestern (β = −0.066), or Northeastern (β = −0.062), Good health status (β = 0.094), and higher self-rated knowledge levels (all *p* < 0.001) were significant predictors.

**Conclusion:**

These findings reveal significant gaps in knowledge and practice among university students. They provide a critical evidence base for developing targeted curricular interventions and health promotion strategies within higher education to improve occupational health literacy.

## 1 Introduction

Pneumoconiosis remains a severe global public health burden, particularly in low- and middle-income countries. While age-standardized incidence and mortality have declined, the absolute number of cases continues to rise, with China bearing a disproportionate share of this burden (1–3). This irreversible occupational lung disease, caused by prolonged inhalation of dust and leading to pulmonary fibrosis (4–6), substantial disability, comorbidity, and reduced quality of life (7–11). Although national rehabilitation initiatives have been launched globally (12–14), the ongoing emergence of new cases underscores the critical limitation of a treatment-focused approach and highlights prevention as the paramount strategy (15). Relying solely on treatment and rehabilitation can only control the condition, prevent deterioration, and alleviate symptoms, but cannot reduce the incidence of the disease (2, 16). Reducing the intensity and frequency of exposure to risk and implementing effective prevention measures are necessary.

Despite the importance of prevention, current policies, research, and interventions narrowly target on-site occupational groups, such as miners and manufacturing workers (17–21). University students represent a critical yet understudied group in the continuum of occupational health prevention. As future members of the workforce and potential policymakers, their knowledge, attitudes, and preventive competencies regarding occupational diseases will shape the effectiveness of workplace health practices and policy implementation. Moreover, the university stage is a formative period for developing health beliefs and behavioral norms (22). The Knowledge–Attitude–Practice (KAP) model, a mainstay of health behavior science, frames the pathway from knowledge acquisition to attitude formation and, ultimately, to practice. While widely applied in other health domains such as patient self-management, education and training, and nursing management (23–26), its application to evaluate pneumoconiosis-related KAP in a pre-employment, non-occupational cohort represents a novel contribution.

Here, we conducted the first large-scale nationwide cross-sectional study to assess KAP regarding pneumoconiosis prevention among Chinese university students and the associated demographic and social factors. Our specific objectives were threefold: (1) to benchmark their current KAP levels; (2) to identify key demographic and social determinants of KAP levels; and (3) to generate evidence foundations for designing targeted curricular interventions that embed occupational health literacy into higher education curricula.

## 2 Materials and methods

### 2.1 Study design and participants

We conducted a nationwide cross-sectional online survey between September and October 2025. Using stratified random sampling by educational levels and geographic regions, we recruited 1,795 students from 84 universities across 33 Chinese provinces. Inclusion criteria were current university enrollment and provision of informed consent. Prior to the online survey, all participants provided informed consent through the questionnaire instructions. The study was approved by the Medical Ethics Committee of Wuhan Polytechnic University (Approval No. BME-2025-1-23) (see Supplementary Appendix I).

### 2.2 Questionnaire development and validation

A questionnaire was developed based on the Knowledge-Attitude-Practice (KAP) theoretical framework, informed by literature review (27–33) and expert consultation. Following two rounds of expert consultation and a pilot test (*n* = 50), items with correlation coefficients <0.4 were removed. These 50 pilot participants were excluded from the final analysis. The final scale contains 45 items: 17 knowledge items, 14 attitude items, and 14 practice items (final questionnaire see the Supplementary Appendix II). All items were scored on a 5-point Likert scale. Instrument validation in the pilot sample showed excellent internal consistency (Cronbach’s α: overall = 0.925, knowledge = 0.954, attitude = 0.785, practice = 0.902). The Kaiser-Meyer-Olkin measure was 0.787, indicating sampling adequacy for factor analysis. In this study, raw scores for each dimension (knowledge: 17–85; attitude: 14–70; behavioral intention: 14–70) were converted to standardized scores using the formula: (actual score ÷ maximum possible score) × 100. Standardized scores were categorized as low (<60), moderate (60–85), or high (>85) (34) based on established conventions in KAP research.

### 2.3 Data collection and quality control

Participants completed the anonymous online questionnaire via the WenJuanXing platform (35, 36). To ensure data quality: (1) the survey was administered anonymously; (2) all scale items were mandatory, and each IP address was restricted to a single submission. (3) standardized instructions with key term definitions were provided; and (4) quality-control items were embedded. All scale items were set as mandatory on the WenJuanXing platform. Consequently, no missing data were present in any of the retained 1,795 questionnaires. Questionnaires with any missing responses were excluded during quality control screening. Two researchers independently screened all responses and excluded questionnaires with completion times <50% of the sample mean, illogical or nconsistent responses, incorrect attention-check responses, or evidence of patterned responding.

### 2.4 Sampling and statistical analysis

An *a priori* sample size was calculated using G*Power 3.1.9.4 (effect size *f*^2^ = 0.02, α = 0.05, power = 0.95, 13 predictors), resulting in a sample of 1,339 participants. Allowing for 10% invalid responses, the target sample size was 1,473.

Data were analyzed using SPSS 25.0. Continuous variables are presented as mean ± standard deviation (SD), and categorical variables as frequencies and percentages. Between-group comparisons were conducted using one-way ANOVA. The following independent variables were included in the analysis: gender (1 = male, 2 = female), Nation (1 = The Han nationality, 2 = The non-Han nationality), major (1 = Medical-related disciplines, 2 = Non-Medical-related disciplines), current education level (1 = Associate degree, 2 = Undergraduate, 3 = Master’s degree and above), year of study (1 = 1st year, 2 = 2nd year, 3 = 3rd year, 4 = 4th year), university region (1 = Eastern China, 2 = Southern China, 3 = Central China, 4 = Northern China, 5 = Northwestern China, 6 = Southwestern China, 7 = Northeastern Chin), place of habitual residence (1 = urban, 2 = County-level city, 3 = Township, 4 = Rural area), parental education level (1 = <Associate degree, 2 = ≥Associate degree), self-reported academic performance (1 = Excellent, 2 = Average, 3 = Poor), household income level (1 = low, 2 = Medium, 3 = High), self-rated health status (1 = poor, 2 = Average, 3 = Good), and self-perceived knowledge level (1 = very unfamiliar, 2 = unfamiliar, 3 = Average, 4 = familiar, 5 = very familiar). Factors associated with KAP scores were identified using multiple linear regression. All variables with *p* < 0.05 in the univariate analysis were entered as independent variables into a multiple linear regression model (enter method) to identify factors independently associated with overall KAP scores. Statistical significance was set as *p* < 0.05 (two-tailed).

## 3 Results

### 3.1 Participant characteristics

A total of 2,002 university students from 84 institutions across 33 Chinese provinces were initially invited to participate via the Wenjuanxing online platform. After excluding invalid responses, 1,795 valid questionnaires were retained, yielding an effective response rate of 89.66%. Participants were aged 19.28 ± 1.95 years (range: 15–25 years). Ethnic minorities (i.e., Non-Han nationality) constituted 13.43%, while fourth-year undergraduates (0.95%) and postgraduate students (2.28%) represented smaller proportions (see Table 1).

**Table 1:** Baseline of participants and univariate associations with KPA scores (*n* = 1,795).

Variables		*n* (%)	KAP score (mean ± SD)	*F*/*t*	*P*
Gender	Male	355 (19.8)	151.49 ± 25.86	0.742	0.458
	Female	1,440 (80.2)	150.37 ± 23.80		
Nation	The Han nationality	1,554 (86.6)	151.61 ± 24.24	4.536	0.000
	Non-Han nationality	241 (13.4)	144.05 ± 22.99		
Father’s educational level	<Associate degree	1,569 (87.4)	149.79 ± 24.03	−3.705	0.000
	≥Associate degree	226 (12.6)	156.15 ± 24.78		
Mother’s educational level	<Associate degree	1,608 (89.6)	150.13 ± 24.03	−2.412	0.016
	≥Associate degree	187 (10.4)	154.63 ± 25.45		
Major	Medical-related disciplines	1,349 (75.2)	152.35 ± 24.32	5.401	0.000
	Non-medical disciplines	446 (24.8)	145.27 ± 23.12		
Year of study	Year 1	932 (51.9)	151.96 ± 24.88	7.469	0.000
	Year 2	633 (35.3)	147.10 ± 22.90		
	Year 3	213 (11.9)	154.26 ± 23.66		
	Year 4	17 (0.9)	156.82 ± 27.03		
University region	Eastern China	665 (37.0)	155.36 ± 24.9	8.318	0.000
	Southern China	88 (4.9)	147.85 ± 25.53		
	Central China	252 (14.0)	148.8 ± 23.33		
	Northern China	173 (9.6)	151.8 ± 23.75		
	Northwestern China	207 (11.5)	146.36 ± 23.02		
	Southwestern China	278 (15.5)	145.96 ± 21.67		
	Northeastern China	132 (7.4)	146.69 ± 25.04		

Variables		*n* (%)	KAP score (mean ± SD)	*Z*/*t*	*P*
Household income level	Low	267 (14.9)	146.35 ± 25.66	12.863	0.000
	Medium	776 (43.2)	148.91 ± 23.20		
	High	752 (41.9)	153.83 ± 24.33		
Self-reported academic performance	Excellent	597 (33.3)	151.53 ± 23.66	3.954	0.019
	Average	893 (49.7)	151.17 ± 24.37		
	Poor	305 (17.0)	147.07 ± 24.60		
Current education level	Associate degree	1,106 (61.6)	151.89 ± 25.06	4.259	0.014
	Undergraduate	648 (36.1)	148.60 ± 22.46		
	Master’s degree and above	41 (2.3)	146.95 ± 25.62		
Self-perceived knowledge level	Very familiar	85 (4.7)	186.89 ± 24.45	207.516	0.000
	Familiar	321 (17.9)	167.66 ± 23.99		
	Average	760 (42.3)	150.92 ± 18.46		
	Unfamiliar	551 (30.7)	137.58 ± 18.04		
	Very unfamiliar	78 (4.3)	129.60 ± 24.71		
Self-rated health status	Good	870 (48.5)	154.85 ± 26.12	29.097	0.000
	Average	649 (36.2)	147.62 ± 21.29		
	Poor	276 (15.4)	144.14 ± 21.93		
Place of habitual residence	Urban	1,115 (62.1)	151.89 ± 24.41	3.080	0.027
	County-level city	316 (17.6)	147.61 ± 24.35		
	Township	159 (8.9)	149.45 ± 22.2		
	Rural area	205 (11.4)	149.02 ± 24.06		

### 3.2 KAP scores

As shown in Table 1, the mean overall KAP score was 66.93 ± 10.76, reflecting a low-to-moderate level across the assessed domains. Among the three dimensions, attitude was highest (72.45 ± 9.92), followed by practice (67.65 ± 13.53) and knowledge domains (61.79 ± 16.75). Only 6.85% of participants achieved high overall scores (>85), whereas the majority (67.52%) fell into the moderate range (60–85). Detailed score distributions for each dimension are presented in Table 2.

**Table 2:** KAP scores and classification levels of pneumoconiosis prevention and control among university students (*n* = 1,795).

Variables	Questionnaire score (mean ± SD)		Degree division [*n*(%)]		
	Total score	Standard score	Low range	Medium range	High range
Knowledge	52.52 ± 14.23	61.79 ± 16.75	757 (42.17)	899 (50.08)	139 (7.74)
Attitude	50.72 ± 6.94	72.45 ± 9.92	101 (5.63)	1,543 (85.96)	151 (8.41)
Practice	47.36 ± 9.47	67.65 ± 13.53	334 (18.61)	1,264 (70.42)	197 (10.97)
Total	150.59 ± 24.21	66.93 ± 10.76	460 (25.63)	1,212 (67.52)	123 (6.85)

### 3.3 Lowest-scoring items by dimension

Item-level analysis revealed specific knowledge, attitude, and practice gaps (see Table 3). In the knowledge dimension, participants had limited understanding of pneumoconiosis pathogenesis (2.90 ± 0.99), its incurability (2.91 ± 1.06), and relevant legal protections (2.94 ± 1.01). Concerning the attitude dimension, some students prioritized economic development over workers’ health (2.09 ± 0.98), placed primary responsibility for prevention on workers (2.37 ± 1.04), or considered pneumoconiosis irrelevant to students (2.43 ± 0.88). Practice engagement was lowest for health information dissemination (2.87 ± 1.04), initiating discussions (2.88 ± 0.94), and proactive information seeking (3.07 ± 0.84).

**Table 3:** The three lowest-scoring items in three KAP dimensions among university students (*n* = 1,795).

Variables	Items	Score (mean ± SD)
Knowledge	Do you know the fundamental cause of pneumoconiosis (pulmonary fibrosis)	2.90 ± 0.99
	Do you know that pneumoconiosis is currently incurable	2.91 ± 1.06
	Do you know that there are specific laws in China (such as the “Law on the Prevention and Control of Occupational Diseases”) to protect the health rights of workers exposed to dust	2.94 ± 1.01
Attitude	I hold that, to some extent, compromising the health of certain workers is an inevitable trade-off for economic development	2.09 ± 0.98
	I believe that the prevention of pneumoconiosis primarily rests with workers themselves	2.37 ± 1.04
	I perceive pneumoconiosis as a health issue that is largely remote from the university student population	2.43 ± 0.88
Practice	I have disseminated basic knowledge about pneumoconiosis to my classmates	2.87 ± 1.04
	I engage in discussions about pneumoconiosis-related topics with classmates, friends, or family members	2.88 ± 0.94
	I proactively seek information on occupational diseases/pneumoconiosis through the news media, the internet, and other channels	3.07 ± 0.84

### 3.4 Factors associated with KAP

Univariate analysis identified several demographic and social factors significantly associated with KAP scores (see Table 1). No significant gender differences were observed (*p* > 0.05). However, nation, major, current education level, year of study, university region, place of habitual residence, parental education level, self-reported academic performance, household income level, self-rated health status, and self-perceived knowledge all showed statistically significant associations with KAP scores (all *p* < 0.05).

### 3.5 Multivariate analysis of predictors

Multiple linear regression analysis (Table 4) revealed that Non-Han status (β = −0.072, *p* = 0.001), Non-Medical disciplines (β = −0.062, *p* = 0.005), year of study 3 (β = 0.066, *p* = 0.006), residence in the Northern (β = −0.066, *p* = 0.015), Northwestern (β = −0.073, *p* = 0.005), Southwestern (β = −0.066, *p* = 0.006), or Northeastern (β = −0.062, *p* = 0.003), Good health status (β = 0.094, *p* = 0.001), and higher self-rated knowledge levels (all *p* < 0.001) were significant predictors of KAP.

**Table 4:** Multivariate linear regression predictors of KAP for pneumoconiosis prevention (*n* = 1,795).

Variables	Estimate (B)	Std. error (SE)	Std. estimate (β)	*t*	*P*	95%CI	Tolerance	VIF
(Constant)	128.342	3.135		40.945	0.000	(122.194, 134.49)		
The Han nationality	Ref							
Non-Han nationality	−5.120	1.567	−0.072	−3.268	0.001	(−8.192, −2.047)	0.752	1.330
Associate degree	Ref							
Undergraduate	1.073	1.237	0.021	0.868	0.386	(−1.352, 3.498)	0.608	1.646
Master’s degree and above	4.410	3.254	0.027	1.356	0.175	(−1.971, 10.792)	0.908	1.102
Medical-related disciplines	Ref							
Non-medical disciplines	−3.488	1.254	−0.062	−2.781	0.005	(−5.948, −1.028)	0.730	1.370
Year 1	Ref							
Year 2	−1.085	1.226	−0.021	−0.885	0.376	(−3.49, 1.319)	0.625	1.600
Year 3	4.947	1.781	0.066	2.777	0.006	(1.453, 8.440)	0.646	1.547
Year 4	2.624	5.044	0.010	0.520	0.603	(−7.269, 12.516)	0.899	1.113
East China	Ref							
South China	0.311	2.415	0.003	0.129	0.897	(−4.425, 5.048)	0.789	1.268
Central China	−1.184	1.675	−0.017	−0.707	0.480	(−4.468, 2.100)	0.634	1.578
North China	−5.454	2.231	−0.066	−2.444	0.015	(−9.830, −1.078)	0.495	2.022
Northwest China	−5.521	1.982	−0.073	−2.785	0.005	(−9.409, −1.634)	0.530	1.885
Southwest China	−4.445	1.604	−0.066	−2.770	0.006	(−7.591, −1.298)	0.636	1.571
Northeast China	−5.758	1.909	−0.062	−3.017	0.003	(−9.502, −2.015)	0.864	1.157
Mother’s education < associate degree	Ref							
Mother’s education ≥ associate degree	1.161	1.579	0.015	0.735	0.462	(−1.936, 4.259)	0.921	1.085
Rural area	Ref							
Township	0.650	2.124	0.008	0.306	0.760	(−3.517, 4.817)	0.589	1.699
County-level	−1.960	1.787	−0.031	−1.097	0.273	(−5.464, 1.544)	0.463	2.159
Urban	1.080	1.525	0.022	0.708	0.479	(−1.91, 4.07)	0.392	2.552
Household income low	Ref							
Medium	1.525	1.417	0.031	1.076	0.282	(−1.255, 4.304)	0.435	2.300
High	1.821	1.502	0.037	1.213	0.225	(−1.124, 4.766)	0.390	2.563
Self-report academic performance poor	Ref							
Average	0.983	1.332	0.020	0.738	0.461	(−1.629, 3.595)	0.483	2.070
Excellent	0.577	1.422	0.011	0.406	0.685	(−2.212, 3.365)	0.478	2.093
Health status poor	Ref							
Average	2.314	1.437	0.046	1.610	0.107	(−0.504, 5.133)	0.450	2.224
Good	4.532	1.423	0.094	3.185	0.001	(1.741, 7.322)	0.424	2.360
Self-perceived knowledge very familiar	Ref							
Familiar	7.482	2.389	0.143	3.132	0.002	(2.797, 12.167)	0.177	5.662
Average	20.041	2.362	0.409	8.485	0.000	(15.408, 24.673)	0.157	6.359
Unfamiliar	36.480	2.526	0.577	14.443	0.000	(31.526, 41.434)	0.229	4.369
Very unfamiliar	54.230	3.144	0.476	17.251	0.000	(48.065, 60.396)	0.481	2.079
R2:0.353								
Adjusted R2:0.343								
*F* (27, 1,767) = 35.792, *p* = 0.000								

The regression model explained 35.2% of the total variance in KAP scores (adjusted *R*^2^ = 0.352) and was statistically significant (*F* = 35.792, *p* < 0.001). Variance inflation variance inflation factors ranged from 1.268 to 6.359, indicating no concerning multicollinearity.

## 4 Discussion

### 4.1 Moderate but insufficient KAP of pneumoconiosis prevention and control among university students

Our findings indicate that university students in China have a low-to-moderate KAP level regarding pneumoconiosis prevention and control. The three KAP dimensions were unevenly developed: knowledge was weakest (61.79 ± 16.75), attitude was relatively positive (72.45 ± 9.92), and practice lagged (67.65 ± 13.53). The overall pattern-weak knowledge, acceptable attitudes, and poor practice*—*is consistent with previous KAP studies on occupational health among non-occupational groups, suggesting that favorable attitudes do not automatically translate into adequate knowledge or consistent preventive actions (37–39).

Within the knowledge domain, students demonstrated significant deficiencies in understanding pneumoconiosis pathogenesis—specifically, dust-induced pulmonary fibrosis—as well as the irreversible and incurable nature of the disease. They also lacked awareness of key legal frameworks, including the Occupational Disease Prevention and Control Law. These persistent knowledge gaps point to a systemic deficiency in occupational health education embedded within higher education curricula, a finding corroborated by parallel evidence from practicing occupational workers (40). Although students generally exhibited favorable attitudes toward occupational health, several persistent misconceptions were identified. A subset of participants endorsed inaccurate assertions such as “economic development justifies compromising workers’ health” and “pneumoconiosis is irrelevant to university students.” These views signal a tendency to individualize responsibility for health, a cognitive bias that may undermine students’ capacity—and willingness—to advocate for robust occupational health policies in their future roles as professionals or decision-makers.

In practice, students demonstrated limited behavioral competence, exhibiting notably low performance in key action-oriented competencies—including knowledge dissemination, initiating evidence-informed discussions, and proactive information seeking. This pronounced disconnect between theoretical understanding, attitudinal orientation, and observable behavioral enactment reflects a significant translational gap within the KAP framework—indicating that favorable attitudes have not yet been internalized or operationalized into sustained, contextually appropriate actions.

### 4.2 Predictors of KAP and implications for intervention

Multifactorial analysis showed that Non-Han status, Non-Medical disciplines, residence in Northern, Northwestern, Southwestern, or Northeastern regions, and higher self-rated knowledge levels were the predictors of KAP scores.

Among non-Han students, KAP scores were significantly lower, This finding is consistent with previous research (41, 42). While this disparity may reflect linguistic barriers, cultural contexts, and constrained health information access, smaller subgroup samples (13.43%) limit precise estimation of ethnic differences and call for verification in larger, stratified studies.

The lower scores among non-medical students likely result from the absence of systematic training or course integration on occupational health topics, a pattern consistent with other studies (43–45).

Regional disparities—with students from Northern, Northwestern, Southwestern, and Northeastern China scoring lower than those from Eastern China—may stem from differences in industrial structures, economic development levels, and regional emphasis on occupational health education (46).

At the individual level, better self-rated health was positively associated with KAP scores, suggesting that students who perceive themselves as healthier may be more motivated to acquire occupational health knowledge and adopt preventive practice (47).

A significant dose–response relationship was also observed between students’ self-rated knowledge and their total KAP scores, highlighting that enhancing students’ subjective familiarity with the topic may be a key step for motivating learning and shaping positive attitudes (48–51).

Based on these findings, our study suggests that integrating occupational health education into general higher education curricula could be beneficial. Universities may consider developing modular courses covering fundamental knowledge of occupational disease prevention, identification and control of typical occupational hazards such as dust, and relevant laws and regulations. At the teaching implementation level, student-centered educational strategies—such as interactive workshops, real-world case discussions, and thematic advocacy activities—may help correct misconceptions and promote positive shifts from cognition to behavior. However, we emphasize that these suggestions are preliminary and should be evaluated in future intervention studies.

## 5 Limitations

This study has several limitations. First, the cross-sectional design precludes causal inference between the identified factors and KAP outcomes. We can only describe associations, not determine directionality or causality. Second, the reliance on self-reported data may introduce recall bias (inaccurate recollection of one’s own knowledge or practice) and social desirability bias (over-reporting of favorable attitudes or practices). Third, the use of an online convenience sample via the WenJuanXing platform may introduce selection bias, as students with internet access and willingness to complete online surveys may differ from the general university population. Fourth, the present study did not account for psychological and cognitive mediators (e.g., risk perception, perceived susceptibility, self-efficacy) that may influence the translation of KAP into preventive practice. Future investigations integrating these psychosocial dimensions may yield a more comprehensive understanding of the mechanisms driving occupational health behavior among university students.

## 6 Conclusion

This study demonstrates that Chinese university students possess a moderate level of KAP regarding pneumoconiosis, with significant gaps in knowledge and practice despite relatively positive attitudes. Key determinants of KAP performance include ethnic background, academic discipline, geographic region, self-rated health, and perceived knowledge.

These findings provide preliminary evidence to suggest that integrating occupational health and safety education into higher education curricula may serve as a component of primary prevention. However, causal conclusions and definitive policy recommendations cannot be drawn from this cross-sectional study. Future longitudinal and interventional research is needed to evaluate whether curricular enhancements can effectively improve students’ occupational health competence and translate knowledge into preventive action.

## Data Availability

The datasets presented in this study can be found in online repositories. The names of the repository/repositories and accession number(s) can be found in the article/Supplementary material.
