# Supplementary material for: Knowledge-attitude-practice of pneumoconiosis prevention and control among Chinese university students: a cross-sectional study
Source: Front Public Health. 2026 Jun 24;14:1816856. doi: 10.3389/fpubh.2026.1816856 (PMC13342244; doi:10.3389/fpubh.2026.1816856)
Supplement: Supplementary file 1 [file Table_1.DOCX]

Supplementary Material

# Appendix Ⅰ


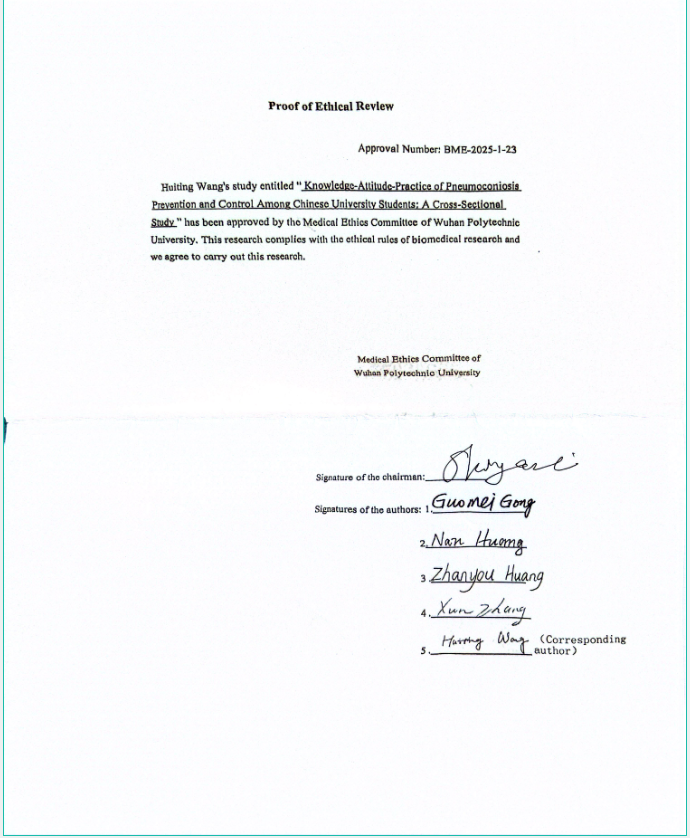


# Appendix Ⅱ

**Investigation on university students' Knowledge-Attitude-Practice Regarding Pneumoconiosis Prevention and control**

Hello!

Thank you for taking the time to assist us with this research topic. This questionnaire aims to understand university students' knowledge, attitudes, and behaviors regarding pneumoconiosis prevention and control. It is solely for scientific research purposes and is conducted anonymously. We will strictly keep the data confidential, and it will not have any impact on you or your family. Please fill it out objectively based on your own situation. Thank you for your cooperation! This form primarily seeks to gather your personal information. Please select the options that best match your circumstances based on the actual situation.

Ⅰ. Basic Information

(Ⅰ) Characteristics of Homo sapiens

1. Your gender [single answer] *

| ○Male |
| --- |
| ○Female |

2. Your age [fill in the blank answers] *

_________________________________

3. Your nation [fill in the blank answers] *

_________________________________

4. The full name of the university you attended is [fill in the blank answers]*

_________________________________

5. Your current education level [Single-choice question]*

| ○Associate degree |
| --- |
| ○Undergraduate |
| ○Master's degree and above |

6. Your Major (Please fill in the full name) [fill in the blank answers] *

_________________________________

7. Which year of study are you currently in [fill in the blank answers]*

_________________________________

8. The main regions you have resided in are: [fill in the blank answers] *

Province:_______________   
City:____________

County (District):____________

9. The region where your university is located: [single answer]*

| ○Eastern China (including Shandong, Jiangsu, Anhui, Zhejiang, Fujian, and Shanghai) |
| --- |
| ○Southern China (including Guangdong, Guangxi, and Hainan) |
| ○Central China (including Hubei, Hunan, Henan, Jiangxi) |
| ○Northern China (including Beijing, Tianjin, Hebei, Shanxi, and Inner Mongolia) |
| ○Northwestern China (including Ningxia, Xinjiang, Qinghai, Shaanxi, and Gansu) |
| ○Southwestern China (including Sichuan, Yunnan, Guizhou, Xizang, and Chongqing) |
| ○Northeastern China (including Liaoning, Jilin, Heilongjiang) |

10. Where did you primarily reside before attending university? [single answer] *

| ○Urban |
| --- |
| ○County-level city |
| ○Township |
| ○Rural area |

(Ⅱ)Socioeconomic factors

11. What is the household income level? (Please make the judgment based on the living standards in your hometown)[single answer] *

| ○Lower income (e.g., can meet basic needs for food, clothing, and education, with stable basic living conditions, but large non-essential expenses still require savings or are difficult to afford)   \| ○ Middle-income (e.g., having disposable income after meeting basic needs, capable of regularly paying for ordinary non-essential expenses) \| \| --- \| \| ○ High income (e.g., financial freedom, ability to simultaneously afford multiple high-quality non-essential expenditures, premium tutoring, easily covering major expenses such as studying abroad, leisure travel, etc.) \| |
| --- | --- | --- |
|  |

12. Your father's educational level [single answer] *

| ○<Associate degree |
| --- |
| ○≥Associate degree |

13. Your mother's educational level [single answer] *

| ○<Associate degree |
| --- |
| ○≥Associate degree |

14. Your self-reported academic performance [single answer] *

| ○Excellent (Ranked in the top 20% of the class) |
| --- |
| ○Average (Ranked in the top 21-40% of the class) |
| ○Poor (Class ranking after 41%) |

(Ⅲ)Health-related factors

15. What is your self-rated health status? [single answer] *

| ○Good |
| --- |
| ○Average |
| ○Poor |

16. What is your self-perceived knowledge level? [single answer] *

| ○Very familiar |
| --- |
| ○Familiar |
| ○Average |
| ○Unfamiliar |
| ○Very unfamiliar |
| 17. Your channels for acquiring knowledge related to pneumoconiosis prevention and control or other scientific information [Multiple Choice] *  □Parents, friends, or other Homo sapiens |
| □Courses or related lectures |
| □Health Promotion Brochure/Leaflet |
| □Hospital physical examination |
| □network platform |
| □Community Promotion |
| □Government presentation |
| □media publicity |
| □Other channels _________________ |
| □unknown |
| 18. In the future, through what channels do you hope to acquire knowledge related to pneumoconiosis prevention and control? [Multiple Choice] *  □Self-learning with the aid of AI platforms |
| □Popular Science Knowledge Lecture on Campus |
| □Hands-on Scientific Experiments |
| □Interesting Science Popularization Graphics and Text |
| □Popular Science Video |
| □newspapers [papers] and periodicals [magazines] |
| □others_________________ |

Instructions: The following is a knowledge, attitude, and practice (KAP) survey consisting entirely of single-choice questions. Please patiently and truthfully complete it by selecting the option that best reflects your personal homo sapiens perspective. Do not omit any responses. Thank you for your cooperation!

II. Knowledge Section

19. Please select your answer based on your understanding [Matrix single answer]*

|  | No Knowledge at All | Limited Knowledge | Moderate Knowledge | Good Knowledge | Expert Knowledge |
| --- | --- | --- | --- | --- | --- |
| 1  Do you know that pneumoconiosis is a legally recognized occupational disease? | 1. ○ | ○ | ○ | ○ | ○ |
| 2  Do you know what the main pathogenic factors (pathogenic dust) causing pneumoconiosis are? | ○ | ○ | ○ | ○ | ○ |
| 3  Do you know the fundamental cause of pneumoconiosis (pulmonary fibrosis)? | ○ | ○ | ○ | ○ | ○ |
| 4  Do you know that pneumoconiosis is currently incurable? | ○ | ○ | ○ | ○ | ○ |
| 5  Do you know the main clinical symptoms of pneumoconiosis (such as coughing, chest tightness, and breathing difficulties)? | ○ | ○ | ○ | ○ | ○ |
| 6  Do you know that pneumoconiosis can lead to various serious complications (such as tuberculosis, pneumothorax, and pulmonary heart disease)? | ○ | ○ | ○ | ○ | ○ |
| 7  Do you know which industries or occupations are at high risk of dust exposure (such as mining, stone processing, and tunnel construction)? | ○ | ○ | ○ | ○ | ○ |
| 8  Do you know that there are specific laws in China (such as the "Law on the Prevention and Control of Occupational Diseases") to protect the health rights of workers exposed to dust? | ○ | ○ | ○ | ○ | ○ |
| 9  Do you know that employers must provide effective protective equipment (such as dust masks) to workers exposed to dust? | ○ | ○ | ○ | ○ | ○ |
| 10  Do you know that ordinary medical masks have limited effectiveness in protecting against production dust? | ○ | ○ | ○ | ○ | ○ |
| 11  Do you know that the diagnosis of pneumoconiosis must be conducted by a legally recognized occupational disease diagnosis institution? | ○ | ○ | ○ | ○ | ○ |
| 12  Do you know that pneumoconiosis patients are entitled to work-related injury insurance benefits by law? | ○ | ○ | ○ | ○ | ○ |
| 13  Do you know that the concentration of dust and the duration of exposure are key factors influencing the occurrence of pneumoconiosis? | ○ | ○ | ○ | ○ | ○ |
| 14  Do you know that "occupational health examinations" are an important means of detecting early-stage pneumoconiosis? | ○ | ○ | ○ | ○ | ○ |
| 15  Do you know that all types of school-run factories or workshops in middle and primary schools are prohibited from engaging in dust-generating operations? | ○ | ○ | ○ | ○ | ○ |
| 16  Do you know that whole lung lavage for pneumoconiosis is not a cure but a symptomatic treatment? | ○ | ○ | ○ | ○ | ○ |
| 17  Do you know that under the same working conditions, workers with poorer physical fitness have a higher probability of contracting the disease? | ○ | ○ | ○ | ○ | ○ |

Ⅲ. Attitude part

20. Please select your answer based on your understanding.[Matrix single answer]*

|  | Strongly Disagree | Disagree | Neutral | Agree | Strongly Agree |
| --- | --- | --- | --- | --- | --- |
| 1  I perceive pneumoconiosis as a health issue that is largely remote from the university student population. | ○ | ○ | ○ | ○ | ○ |
| 2  I believe that the prevention of pneumoconiosis primarily rests with workers themselves. | ○ | ○ | ○ | ○ | ○ |
| 3  I hold that, to some extent, compromising the health of certain workers is an inevitable trade-off for economic development. | ○ | ○ | ○ | ○ | ○ |
| 4  I maintain that university students bear a responsibility to learn about and engage with social issues such as pneumoconiosis. | ○ | ○ | ○ | ○ | ○ |
| 5  I contend that the media should significantly intensify its coverage of occupational diseases, including pneumoconiosis. | ○ | ○ | ○ | ○ | ○ |
| 6  If my future occupation involves exposure to airborne dust, I would experience substantial concern regarding my long-term health. | ○ | ○ | ○ | ○ | ○ |
| 7  I am willing to participate in volunteer service activities aimed at supporting individuals affected by pneumoconiosis. | ○ | ○ | ○ | ○ | ○ |
| 8  I consider enhancing industrial processes—thereby reducing dust generation at the source—to be more effective than relying solely on individual protective measures. | ○ | ○ | ○ | ○ | ○ |
| 9  I believe public awareness of pneumoconiosis in contemporary society remains critically insufficient. | ○ | ○ | ○ | ○ | ○ |
| 10  Should a family member or friend work in a high-risk occupation, I would proactively advise them on appropriate protective measures. | ○ | ○ | ○ | ○ | ○ |
| 11  I view attention to and improvement of pneumoconiosis-related conditions as an essential component of the social responsibility expected of today's youth. | ○ | ○ | ○ | ○ | ○ |
| 12  I am prepared to support pneumoconiosis-related public welfare initiatives through volunteer service and other forms of civic engagement. | ○ | ○ | ○ | ○ | ○ |
| 13  I regard employers' deliberate failure to provide adequate dust-control measures—despite awareness of associated risks—as tantamount to criminal negligence. | ○ | ○ | ○ | ○ | ○ |
| 14  I consider acquiring knowledge about pneumoconiosis and adopting preventive practices to be integral to responsible career planning and personal professional development. | ○ | ○ | ○ | ○ | ○ |

Ⅳ. Practice part

21. Please select your answer based on your actual situation.[Matrix single answer]*

|  | Never | Rarely | Sometimes | Often | Always |
| --- | --- | --- | --- | --- | --- |
| 1  I proactively seek information on occupational diseases/pneumoconiosis through the news media, the internet, and other channels. | ○ | ○ | ○ | ○ | ○ |
| 2  When encountering news reports or documentaries about pneumoconiosis, I actively click and read/watch them in detail. | ○ | ○ | ○ | ○ | ○ |
| 3I engage in discussions about pneumoconiosis-related topics with classmates, friends, or family members. | ○ | ○ | ○ | ○ | ○ |
| 4  When exposed to dusty environments (e.g., renovation work), I proactively wear protective masks. | ○ | ○ | ○ | ○ | ○ |
| 5  When selecting internships or employment, I consider whether the position involves occupational disease hazards. | ○ | ○ | ○ | ○ | ○ |
| 6  I remind relatives and friends working in potentially dusty environments to pay attention to occupational protection. | ○ | ○ | ○ | ○ | ○ |
| 7  Upon learning about charitable organizations assisting pneumoconiosis patients, I follow their official accounts. | ○ | ○ | ○ | ○ | ○ |
| 8  I have disseminated basic knowledge about pneumoconiosis to my classmates. | ○ | ○ | ○ | ○ | ○ |
| 9  If I suspect exposure to hazardous dust, I proactively consult relevant materials or seek medical advice. | ○ | ○ | ○ | ○ | ○ |
| 10  When observing workers at nearby construction sites or factories not wearing protective masks, I recognize this as high-risk behavior. | ○ | ○ | ○ | ○ | ○ |
| 11  I have participated in social welfare activities through school clubs or student organizations. | ○ | ○ | ○ | ○ | ○ |
| 12  I am clearly aware of how to access relevant laws and regulations, such as the Occupational Disease Prevention and Control Law. | ○ | ○ | ○ | ○ | ○ |
| 13  If I become a manager in the future, I will prioritize employees' occupational health and safety. | ○ | ○ | ○ | ○ | ○ |
| 14  I am willing to invest time in learning how to properly select and wear personal protective equipment. | ○ | ○ | ○ | ○ | ○ |

22. Do you have any other suggestions regarding pneumoconiosis that you would like to mention? Or any thoughts you wish to express? [fill in the blank answers]

_________________________________
